# Alevin efficiently estimates accurate gene abundances from dscRNA-seq data

**DOI:** 10.1186/s13059-019-1670-y

**Published:** 2019-03-27

**Authors:** Avi Srivastava, Laraib Malik, Tom Smith, Ian Sudbery, Rob Patro

**Affiliations:** 1 Department of Computer Science, Stony Brook University, Stony Brook, USA; 2 Cambridge Centre for Proteomics, Department of Biochemistry, University of Cambridge, Cambridge, CB2 1GA UK; 3 Sheffield Institute for Nucleic Acids, Department of Molecular Biology and Biotechnology, The University of Sheffield, Sheffield, S10 2TN UK

**Keywords:** Single-cell RNA-seq, UMI deduplication, Quantification, Cellular barcode

## Abstract

We introduce alevin, a fast end-to-end pipeline to process droplet-based single-cell RNA sequencing data, performing cell barcode detection, read mapping, unique molecular identifier (UMI) deduplication, gene count estimation, and cell barcode whitelisting. Alevin’s approach to UMI deduplication considers transcript-level constraints on the molecules from which UMIs may have arisen and accounts for both gene-unique reads and reads that multimap between genes. This addresses the inherent bias in existing tools which discard gene-ambiguous reads and improves the accuracy of gene abundance estimates. Alevin is considerably faster, typically eight times, than existing gene quantification approaches, while also using less memory.

**Electronic supplementary material:**

The online version of this article (10.1186/s13059-019-1670-y) contains supplementary material, which is available to authorized users.

## Background

There has been a steady increase in the throughput of single-cell RNA-seq (scRNA-seq) experiments, with droplet-based protocols (dscRNA-seq) [1–3] facilitating experiments assaying tens of thousands of cells in parallel. The three most widely used dscRNA-seq protocols: Drop-seq [1], inDrop [2], and 10X Chromium [3], use two separate barcodes that require appropriate processing for accurate quantification estimation. First, cellular barcodes (CBs) are used to tag each cell with a unique barcode, which enables pooling of cells for sequencing and their subsequent separation in silico. Thus, data processing requires the identification of the true CBs corresponding to distinct cells, and grouping the reads accordingly. Second, identification of PCR duplicates is aided by unique molecular identifiers (UMIs), which tag each unique molecule prior to amplification. Since the mRNA capture rate is only around 5–10% [4], many rounds of PCR are typically performed prior to sequencing [1]. Appropriately accounting for the barcode information is therefore crucial for accurate estimation of gene expression. Only a minor fraction of the possible CBs present will ultimately tag a cell, and likewise, only a minor fraction of UMIs will tag unique molecules from the same gene. Thus, in each case, the aim is to identify the barcodes used. Unfortunately, both CBs and UMIs are subject to errors that occur during sequencing and amplification [1, 5], which makes the accurate deconvolution of this information in silico a non-trivial task. This task is made more difficult by the amplification of background RNA from empty droplets (ambient CBs) or damaged cells.

Various methods have been proposed to correctly process dscRNA-seq barcodes in an error-aware manner (“whitelisting”) [3, 5–8], to correct sequencing errors in CBs and UMIs [5, 8], to deduplicate UMI tags inferred to be duplicates [5], and to obtain cell-level gene quantification estimates [9]. Here, we describe an end-to-end quantification pipeline that takes as input sample-demultiplexed FASTQ files and outputs gene-level UMI counts for each cell in the library. We call this unified pipeline alevin, and it overcomes two main shortcomings of traditional pipelines. First, existing techniques for UMI deduplication discard reads that map to more than one gene. In bulk RNA-seq datasets (with paired-end reads and full-length transcript coverage), the proportion of gene-ambiguous reads is generally small (Table 1). Yet, in tagged-end scRNA-seq, this set of gene-ambiguous reads is generally larger and commonly accounts for ∼ 14–23% of the input data (Table 2). This is a result of both the fact that dscRNA-seq protocols, by construction, display a very strong 3 ^′^ bias and that these protocols yield effectively single-end reads (only one of the sequenced reads contains sequence from the underlying transcript), resulting in a reduced ability to resolve multimapping using a pair of reads from a longer fragment. We show that discarding the multimapping reads can negatively bias the gene-level counts predicted by various methods. Second, existing quantification pipelines combine independent processing algorithms and tools for each step, usually communicating results between pipeline stages via intermediate files on disk, which significantly increases the processing time and memory requirements for the complete analysis. We show that alevin makes use of more reads than other pipelines, that this leads to more accurate quantification of genes, and that alevin does this ∼ 8 times faster and with a lower memory requirement, when compared to existing best practice pipelines for dscRNA-seq analysis.

**Table 1:** The percentage of reads multimapping in bulk datasets from human and mouse

Species	Accession number	Read length	Percentage
Human	SRR1303990 [32]	101	7.4
Human	SRR1373442 [33]	49	9.2
Human	SRR1644186 [34]	100	9.2
Human	SRR5074291 [35]	150	7.7
Mouse	ERR435943 [36]	75	23
Mouse	SRR3532922 [37]	125	10.6
Mouse	SRR6753775 [38]	150	5.6
Mouse	SRR327047 [22]	120	5.2

**Table 2:** Percentage of reads multimapping across various scRNA-seq samples, using the alevin mappings

Sample	Percentage
Human PBMC 4k	14.2
Human PBMC 8k	14.1
Mouse neurons 900	21.8
Mouse neurons 2k	22.7
Mouse neurons 9k	17.2

## Results

### Alevin overview

**Fig. 1:**
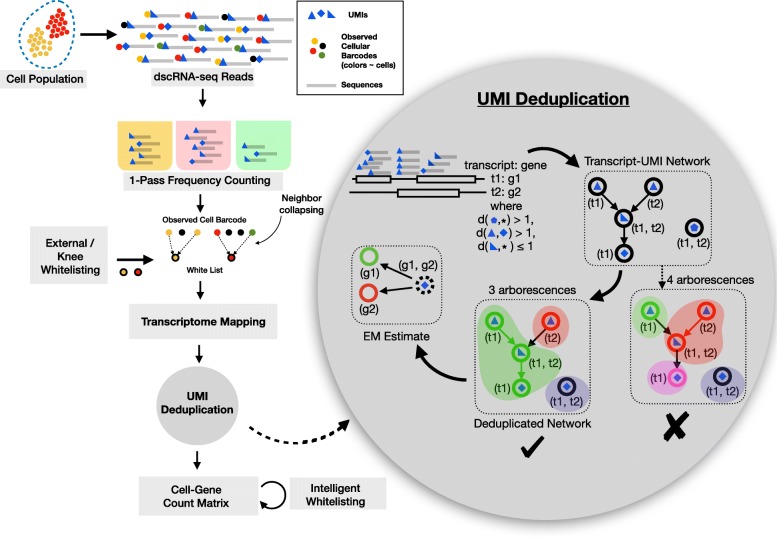
Overview of the alevin pipeline. The input to the pipeline are sample-demultiplexed FASTQ files, and there are several steps, outlined here, that are required to process this data and obtain per-cell gene-level quantification estimates. The first step is cell barcode (CB) whitelisting using their frequencies. Barcodes neighboring whitelisted barcodes are then associated with (collapsed into) their whitelisted counterparts. Reads from whitelisted CBs are mapped to the transcriptome, and the UMI-transcript equivalence classes are generated. Each equivalence class contains a set of transcripts, the UMIs that are associated with the reads that map to each class and the read count for each UMI. This information is used to construct a parsimonious UMI graph (PUG) where each node represents a UMI-transcript equivalence class and nodes are connected based on the associated read counts. The UMI deduplication algorithm then attempts to find a minimal set of transcripts that cover the graph (where each consistently labeled connected component—each monochromatic arborescence—is associated with a distinct pre-PCR molecule). In this way, each node is assigned a transcript label and, in turn, an associated gene label. Reads associated with arborescences that could be consistently labeled by multiple genes are divided amongst these possible loci probabilistically based on an expectation-maximization algorithm. Finally, optionally, and if not provided with high-quality CB whitelist externally, an intelligent whitelisting procedure finalizes a list of high-quality CBs using a naïve Bayes classifier to differentiate between high- and low-quality cells

There are several steps in the alevin pipeline that are streamlined to work without the overhead of writing to disk, as highlighted in Fig. 1 (details in the “Materials and methods” section). The first step is to identify the CBs that represent properly captured and tagged cells (“whitelisting”). Alevin uses a two-step whitelisting procedure, where the second step takes place at the end of the pipeline. An initial whitelist is produced by finding the “knee” in the cumulative distribution of CB frequencies [1, 3]. For each non-whitelisted CB, alevin tries to correct it to a whitelisted CB either by a substitution or by a single insertion or deletion. If no such barcode exists in the set of whitelisted barcodes, the barcode and its associated reads are discarded. The next step is mapping reads from the whitelisted CBs, and the corrected CBs, to a target transcriptome [10, 11], followed by UMI deduplication.

The process of deduplication requires identifying duplicate reads based on their UMIs and alignment positions along the transcriptome. Alevin uses a novel algorithm for deduplication that begins by constructing parsimonious UMI graphs, that we refer to as a PUGs, using information from the UMI sequences, the UMI counts, and the transcript equivalence classes [12]. This PUG is constructed such that each UMI-transcript equivalence class pair is represented by a node and there exists an edge from a node to any node that could have arisen from an amplified molecule due to sampling the underlying transcript (a single pre-PCR molecule) at a different position, or via a PCR or a sequencing error being introduced into the UMI. When the direction of “duplication” during PCR is clear, a directed edge is added; otherwise, a bi-directed edge is placed. An optimal covering of this graph, using the transcripts associated with each node, will give the minimum number of UMIs, along with their counts, required to explain the set of mapped reads. Hence, we have mapped the deduplication problem to that of finding a minimum cardinality covering of a given graph by monochromatic arborescences. Since the decision version of this problem is NP-complete, we propose a greedy algorithm to obtain a minimum cardinality covering of this graph (proof and algorithm detailed under the “Materials and methods” section). Each covering, and the associated UMI, is assigned a set of transcript labels of size ≥ 1. After this UMI resolution phase, the remaining ambiguous reads with more than 1 transcript label are assigned based on an expectation-maximization method [13].

Finally, having obtained per-cell gene expression estimates, CB whitelisting is finalized using a naïve Bayes classifier to differentiate between high- and low-quality cells utilizing a set of features derived from the expression estimates and other diagnostic features [8]. In addition to the gene-by-cell count matrix, alevin also provides information about the reliability of the abundance estimate computed for each gene in each cell in the form of a *tier* matrix (and, optionally, the summarized variance of bootstrap estimates), which succinctly encodes the quality of the evidence used to derive the corresponding count.

### Impact of discarding multimapping reads

**Fig. 2:**
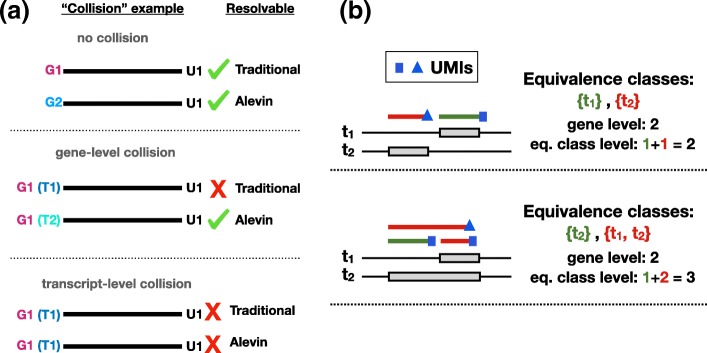
**a** This figure illustrates examples of various classes of UMI collisions and which method(s) would be able to correctly resolve the origin of the multimapping reads in each scenario. These cases are shown top to bottom in order of their likelihood. **b** A simulated example demonstrates how treating equivalence classes individually during UMI deduplication can lead to under-collapsing of UMIs compared to gene-level methods (especially in protocols where the majority of cDNA amplification occurs prior to fragmentation). In the first row, both methods report correctly two UMIs. In the second row, there are two fragmented molecules aligned against two transcripts from the same gene. The alevin deduplication algorithm will attempt to choose the minimum number of transcripts required to explain the read mappings and hence correctly detect the UMI counts. The equivalence class method will over-estimate the gene count

Before proceeding with a more detailed analysis of the alevin pipeline, it is important to highlight scenarios where existing pipelines would fail using simple examples. These also lead to a better understanding of the alevin UMI deduplication algorithm that intelligently utilizes transcript-level information to obtain accurate gene-level estimates. Since current deduplication methods do not have a mechanism to detect UMIs that map between multiple transcripts of the same gene, they can, in certain cases, incorrectly detect PCR duplicates and, hence, under-estimate the total UMI counts. Some obvious cases can be resolved by considering the read-to-transcript mapping, instead of the read-to-gene mapping, as done in alevin and shown in the left panel in Fig. 2. The first row (top to bottom) demonstrates a case when we observe the same UMI (U1) being used to tag transcripts from two separate genes (G1 and G2). Here, all methods are able to correctly assess that these instances of U1 are not PCR duplicates. In the center row, we observe the same UMI deriving from two (sequence-distinct) transcripts of the same gene. Here, purely gene-level methods fail to resolve this collision, while alevin’s strategy can. Finally, in the bottom row, we observe a UMI collision within a single transcript. That is, two different copies (molecules) of the same transcript have been tagged with the same UMI. This cannot be resolved by any of the methods. Though possible, the situation presented in the third row is *highly* unlikely, especially given the current sequencing depths.

A second scenario is highlighted in the right panel of Fig. 2 where using the transcript-level equivalence classes lead to over-counting UMIs (discussed further in the “Materials and methods” section). In these simulated examples, different types of transcripts and corresponding expression patterns are shown. Reads are randomly sampled from the 3 ^′^-end of the annotated transcript(s) according to a realistic fragment length distribution, where exon overlap induces the corresponding equivalence classes of each fragment. The top simulation shows 1 (pre-PCR) molecule expressed for each transcript, identifiable by a unique id (UMI), shown in blue. Due to the disjoint equivalence classes, both methods will correctly assign the gene count. In the bottom simulation, both molecules originate from the second transcript. However, since the equivalence classes are different, the two fragments sharing a UMI will not be collapsed. Specifically, as the rate of splicing (and hence the number of equivalence classes) increases, so too does the number of distinct UMIs reported. In this case, the alevin UMI deduplication algorithm will correctly detect the number of transcripts in order to greedily assign the minimum number of transcripts required to explain the given UMI and mapping information.

**Fig. 3:**
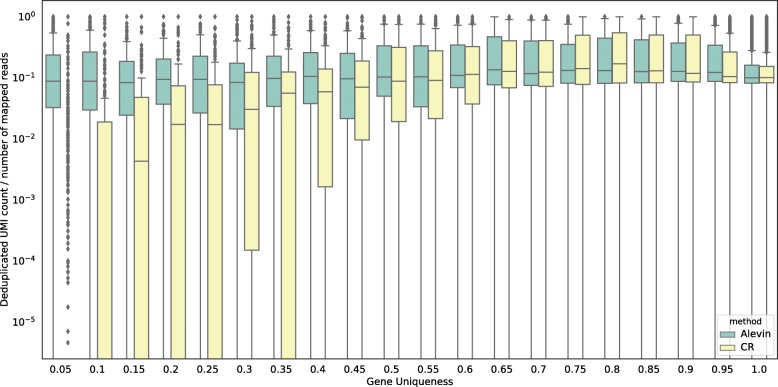
The ratio of the final number of deduplicated UMIs against the number of initial reads for both alevin and Cell Ranger (on the human PBMC 4k dataset) stratified by gene-level sequence uniqueness. The genes are divided into 20 equal sized bins, and the *x*-axis represents the maximum gene uniqueness in each bin. The plotted ratio for genes that have high sequence similarity with other genes is strongly biased when using Cell Ranger. This is because Cell Ranger will discard a majority (or all) of the reads originating from these genes since they will most likely map to multiple positions across various genes. Alevin, on the other hand, will attempt to accurately assign these reads to their gene of origin. This plot also demonstrates that alevin does not over-count UMIs, which would be the case if deduplication was done at the level of equivalence classes

To show that the UMI deduplication algorithm from alevin does, indeed, perform better, we calculate the ratio of the number of reads mapping to each gene and the final count of UMIs as predicted by alevin and Cell Ranger for that gene. When a read maps ambiguously, the count is divided uniformly between the genes. Hence, if a read maps to two genes, the count for each is incremented by 0.5 to get the initial number of reads mapping to these genes. Note that the mappings are also different under each pipeline and that some reads may be inherently ambiguous under one or both mappings. These reads cannot be accurately assigned but, while Cell Ranger discards them, alevin assigns them to a gene via the PUG resolution algorithm, or, in the case that parsimony fails to distinguish a single best gene, proportionally to multiple genes according to the other uniquely mapping reads of the experiment. We divide the genes into 20 bins, based on the number of k-mers shared across genes. We expect the above calculated ratio to remain fairly consistent across these 20 bins, irrespective of the sequence properties of the genes in them. However, we observe in Fig. 3 that the predictions from Cell Ranger are biased for the genes with low sequence uniqueness. This is because a large number of reads from these genes will multimap across genes and will, therefore, be discarded. Hence, simply discarding multimapping reads seems to bias the count estimates for all genes but strongly impacts counts for genes that are expected to have a larger number of multimapping reads due to their high sequence similarity.

### Accuracy analysis on real datasets

To assess the performance of alevin, both in terms of accuracy in quantification and resource consumption, we ran it on 10X Chromium datasets from human and mouse. We compare our results against the Cell Ranger pipeline [3], the dropEst pipeline [8]^1^, and a custom pipeline, with an external list of whitelisted CBs, using STAR [14], featureCounts [15], and UMI-tools [5], which we refer to as the *naïve* pipeline. The exact parameters for running each tool are provided under the “Materials and methods” section. Note that we run alevin with the —keepDuplicates flag during indexing, which ensures that even when multiple sequence-identical transcripts exist in the annotation, they are not discarded. This is to allow for fair comparison against the other tools, since they do not discard such transcripts, and the existence of such transcripts will impact the number of multimapping reads. However, we do not generally recommended using this flag when running alevin. We observe that the number of final whitelisted cells predicted by alevin are in close proximity to the count of cells predicted by Cell Ranger (and dropEst, since they use the same whitelist), but there are non-trivial differences (Table 3). Comparison on data using the Drop-seq [1] protocol is also detailed below. Comparisons against the recently released version 3.0.0 of Cell Ranger are also provided (Additional file 1: Figure S1), along with results from another run of alevin using different parameters. Where mentioned, the results are stratified by gene uniqueness which is the proportion of k-mers, of size 31, that are not shared between two or more genes. We note that varying the k-mer size changes the stratification of the genes but does not impact the overall correlation and performance of the methods. We show this for the mouse neuronal 900 dataset (Additional file 1: Figure S2). We calculated this for each gene in the human (GENCODE release 27, GRCh38.p10) and mouse (GENCODE release M16, GRCm38.p5) transcriptomes. Note that this was not calculated using the canonicalized k-mers from the genes. This is because the scRNA-seq protocols are stranded and a read, therefore, cannot multimap between two genes if the reverse complement of one of them is shared with the other’s forward sequence.

**Table 3:** Number of final whitelisted cellular barcodes output by alevin and Cell Ranger

Dataset	Cell Ranger	Alevin	No. of reads
Human PBMC 4k	4346	4341	379,462,522
Human PBMC 8k	8379	8291	784,064,148
Mouse neurons 900	933	1291	52,805,264
Mouse neurons 2k	2009	1881	147,010,995
Mouse neurons 9k	9116	8519	383,366,284

### Accuracy of estimates against bulk data

To test the accuracy of the quantification estimates, we aggregate the estimates from each of the single-cell quantification tools (summing across all cells) and calculate the correlation with estimates predicted by RSEM [16] (paired with Bowtie2 [17] alignments) using bulk datasets from the same cell types. While the differences between single-cell and bulk sequencing protocols and techniques are significant, we believe that, in the absence of established benchmarks, the correlation between them is a reasonable indicator of the accuracy of each quantification method. Estimates from alevin, when summed across all cells, have a higher Spearman rank correlation than the Cell Ranger, dropEst, and *naïve* pipelines (Table 4). Specifically, we posit that the methods demonstrate a strong and persistent bias against groups of two or more genes that exhibit high sequence similarity. That is, the more sequence-similar a gene is to another gene, the less likely these pipelines are able to assign reads to it—in the extreme case, some genes essentially become *invisible* due to the in silico biases of these approaches (a similar effect was reported by Robert and Watson [18] in bulk RNA-seq data when simple read-counting approaches are used for quantification, where they highlight that many such genes are relevant to human disease).

**Table 4:** Average Spearman correlation of gene-level estimates from each method for the single-cell datasets against bulk data from the same cell types (four for human, three for mouse)

Dataset	Alevin	Cell Ranger	*Naïve*	DropEst
Human PBMC 4k	0.813	0.78	0.747	0.783
Human PBMC 8k	0.81	0.772	0.74	0.776
Mouse neurons 900	0.812	0.773	0.761	0.779
Mouse neurons 2k	0.822	0.781	0.767	0.784
Mouse neurons 9k	0.831	0.796	0.776	0.803

**Fig. 4:**
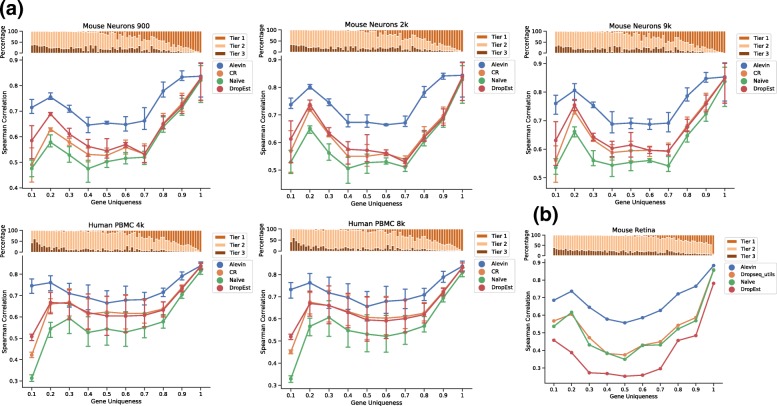
**a** The Spearman correlation between quantification estimates (summed across all cells) from different scRNA-seq methods against bulk data from the mouse neuronal and human PBMC datasets, stratified by gene sequence uniqueness. The bar plot on the top of each figure shows the percentage of genes in each bin that have unique read evidence. Tier 1 is the set of genes with only uniquely mapping reads. Tier 2 is genes that have ambiguously mapping reads, but are connected to unique read evidence that can be used to resolve the multimapping reads. Tier 3 is genes that are completely ambiguous. Note that all methods perform very similarly on genes from tier 1, but the performance of alevin is much better for the other tiers. **b** Comparison of various methods used to process Drop-seq data from mouse retina with 4k cells. The Spearman correlation is calculated against bulk quantification estimates predicted using Bowtie2 and RSEM on data from the same cell type

To further explore this hypothesis, we stratified the accuracy of the different methods by the uniqueness of the underlying genes (Fig. 4a, Table 5). The bar plots at the top of each subfigure represent the tiers of the genes as assigned by alevin. Tier 1 is the set of genes where all the reads are uniquely mapping. Tier 2 is genes that have ambiguously mapping reads, but connected to unique read evidence as well, which can be used by the EM to resolve the multimapping reads. Tier 3 is the genes that have no unique evidence, and the read counts are, therefore, distributed between these genes according to an uninformative prior. In agreement with the hypothesized relationship, we observed that the higher accuracy of alevin is particularly large for genes with a lower proportion of unique k-mers, which tend to belong to tier 2 or 3. On genes from tier 1, all the methods perform similarly. Thus, the approach of Cell Ranger, dropEst, and *naïve*, which discard reads mapping to multiple genes, results in systematic inaccuracies in genes which are insufficiently unique (i.e., which share a high degree of sequence homology with some other gene).

**Table 5:** Number of genes in each bin, when stratified by gene uniqueness

Bin number	Human	Mouse
1	3155	4786
2	894	1089
3	853	945
4	822	1061
5	962	1104
6	1174	1318
7	1565	1476
8	2546	1877
9	4695	2960
10	41622	36763

**Fig. 5:**
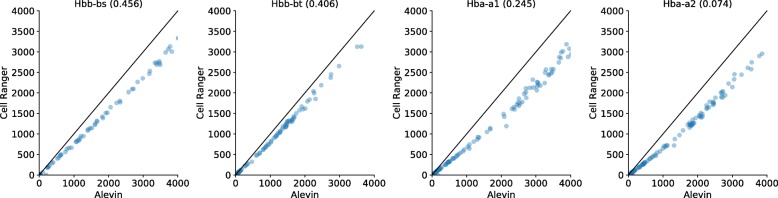
Expression of the Hba and Hbb genes as predicted by alevin and Cell Ranger in mouse neuronal cells. The title of each plot is the name of the gene and its k-mer uniqueness ratio. Note that Cell Ranger systematically under-estimates the expression of these genes compared to alevin. This bias is greater for the Hba genes, which have a lower uniqueness ratio, and therefore, a greater number of multimapping reads

**Fig. 6:**
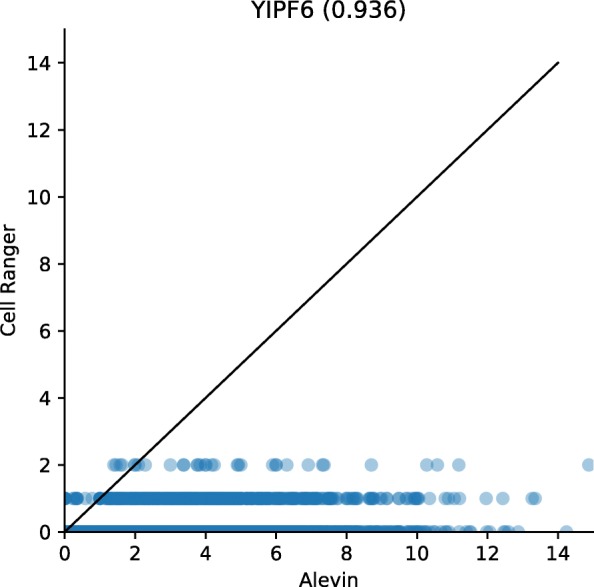
Expression of the YIPF6 gene (which has a high uniqueness ratio) as predicted by alevin and Cell Ranger in the PBMC8k data

This bias could impact the expression estimates of important marker genes, such as the genes for the hemoglobin alpha and beta proteins in the mouse neurons [19, 20]. Due to their lower uniqueness ratio, Cell Ranger appears to exhibit a bias against such genes, and their expression, as predicted by alevin, is systematically higher (Fig. 5). Anecdotally, we also noticed that, in the human PBMC data, alevin sometimes predicts the expression of even relatively sequence-unique genes, like YIPF6, that we expect to be expressed in a subpopulation of these cells (monocytes) [21], but which exhibit almost no expression as predicted by Cell Ranger (Fig. 6). Because the bias against sequence-ambiguous genes is fundamental and sequence-specific, it cannot be easily remedied with more data, but instead requires the development of fundamentally novel algorithms, like alevin, that account for, rather than discard, reads mapping to such genes. Hence, alevin not only quantifies a greater proportion of the sequenced data than existing methods, but also does so more accurately and in a less biased manner.

### Accuracy of estimates using combined genomes

To further assess the accuracy of quantification estimates, in the absence of any established read-level simulation protocol, we performed an experiment aimed to introduce controlled gene-level multimapping to analyze its effect on the different methods. We quantified the mouse neuronal 900 sequencing dataset using both Cell Ranger and alevin, and each quantification was performed under two separate references: the mouse genome and the combined human and mouse genome. Noting that the reads in this experiment originate from mouse, we desire that the quantifications returned by a method deviate as little as possible under the two different reference configurations. Under ideal conditions, for example, the gene counts under both references should be the same. However, combining the mouse and human references increases the gene sequence ambiguity, due to the presence of homologous genes, resulting in misestimation.

**Fig. 7:**
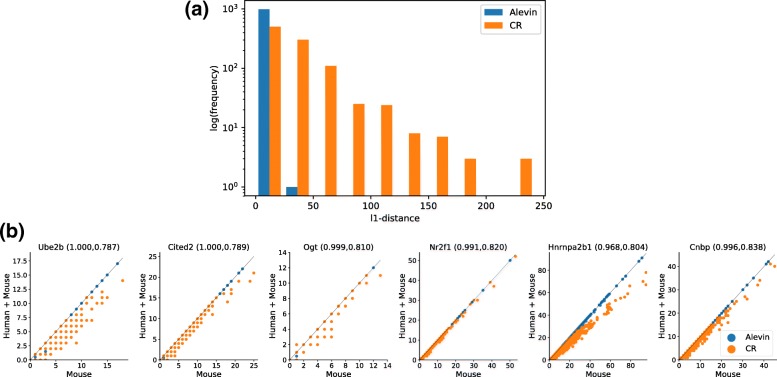
**a** Histogram of the *ℓ*_1_ distance between the quantification estimates of tools on the mouse neuron 900 data, when run using different references for quantification (just mouse versus mouse and human). Results are presented for both alevin and Cell Ranger. Since, in reality, all reads are expected to originate from mouse, deviations from quantifications under the only mouse reference signify misestimation—often due to the introduction of sequence-similar genes in the human genome. Alevin is able to resolve this ambiguity well, while Cell Ranger instead discards such reads, leading to different quantification estimates under the two references. **b** Counts for the topmost genes that have high sequence homology between human and mouse but are sequence unique in the mouse reference. The title of each plot is the gene name along with the sequence uniqueness ratio under just the mouse reference and under the joint reference. Hence, the Cell Ranger counts decrease across cells when the gene uniqueness decreases. Note that these genes were filtered such that they have > 100 count difference for either alevin or Cell Ranger when summed across all cells

We show in Fig. 7a that the distance under the two references is higher for the Cell Ranger estimates than for the alevin estimates. Due to the increased homology among genes between the references, the ratio of reads mapping to multiple genes increases, resulting in more information being discarded by Cell Ranger. The total number of UMIs accounted for by Cell Ranger decreases by ∼ 20,000, in comparison, the number of distinct UMIs predicted by alevin decreased by ∼ 1500, which one might attribute to changes in the underlying PUGs as a result of mapping ∼ 0.01*%* more reads. The number of human genes expressed (non-zero UMI count) under the joint reference is 624 for Cell Ranger and 600 for alevin, out of a total of 58,288 genes. Note that in both cases, these genes account for < 0.05*%* of the total UMI count predicted by each method.

To provide a statistical analysis of the differences observed for the methods under the two different reference sequences, we performed the following test. We sample, randomly, 1000 sets of 100 cells from the entire experiment, and for each sample, we compute the sum of absolute difference between the predictions of each tool under both references. We compare the resulting distribution of differences for Cell Ranger with that of alevin and find that the differences in alevin’s quantifications are smaller than those of Cell Ranger (*p*<0.001, Mann-Whitney-Wilcoxon test). These distributions are plotted in Additional file 1: Figure S3.

We also show in Fig. 7b that, for the genes that have sequence similarity in the joint reference but are unique in the mouse genome, Cell Ranger expression estimates vary much more than those from alevin.

### Time and memory efficiency

**Fig. 8:**
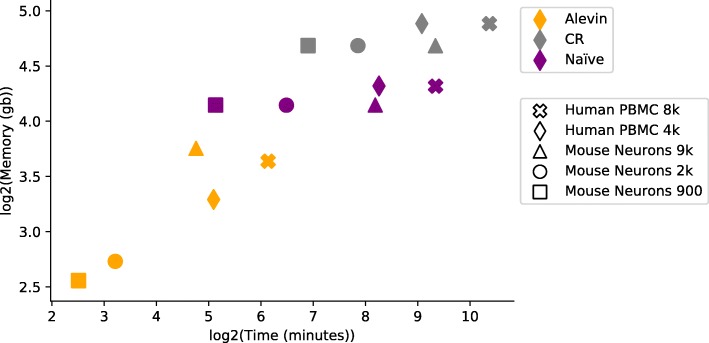
The time and memory performance of the different pipelines on the five datasets. Alevin requires significantly less time and memory than the other pipelines. Note that for Cell Ranger, the memory plotted is the lower bound, which is the size of the index and the actual memory usage can be much higher

The time and memory requirements for alevin are significantly less than those for the existing pipelines (Fig. 8), where all methods were run using 16 threads. DropEst is excluded from the figure since it consumes the BAM file output by Cell Ranger and is not a complete end-to-end pipeline. For the smallest dataset (900 mouse neuronal cells), alevin was ∼ 5 times faster than *naïve* and ∼ 21 times faster than Cell Ranger. This difference increases further as the size of the dataset increases, since the performance of alevin scales better than the other tools. Hence, where alevin took only 70 min to process the human PBMC 8k dataset, Cell Ranger took 22 h and *naïve* took 11 h. On this dataset, dropEst took ∼ 2 hours, after Cell Ranger was used to process and align the reads. In terms of memory, alevin used only ∼ 13 GB on the human PBMC 8k cell dataset, whereas *naïve* took ∼ 20 GB and dropEst took ∼ 32 GB. For the mouse neuronal 9k cell dataset, alevin used ∼ 14 GB, *naïve* ∼ 18 GB, and dropEst ∼ 52 GB. In both cases, Cell Ranger required a minimum of 16 GB just for STAR indexing. We note that Cell Ranger allows the user to specify a maximum resident memory limit, and we ran Cell Ranger allowing it to allocate up to 120 GB so that the extra runtime was not due to limitations in available memory. We also note that for dropEst, we were not able to run the Bayesian collision correction algorithm implemented in dropEstr; however, given the relatively long UMI tags employed in chromium V2 chemistry compared to inDrop, one would expect the effect of this extra phase to be limited anyway.

We observe that the optimal number of threads for running alevin is 10–12, where the maximum gain in terms of time and memory is achieved. Alevin is designed to make efficient use of multiple threads, though the optimal number of threads can depend on many factors, such as the speed of the underlying disk and the size of the raw input and output matrix to be written. While runtime decreases with the number of threads used, the memory profile changes very little as threads are added.

### Comparison on Drop-seq data

In addition to the data generated using the 10X Chromium protocol [3], we also tested alevin on mouse retina data generated using the Drop-seq protocol [1]. We compare alevin against UMI-tools (the *naïve* pipeline from the main paper), dropEst, and dropseq_utils [1]—the processing pipeline originally used by Macosko et al. [1]. Again, we compared the correlation of gene abundances, summed across all cells and as produced by the different methods with the estimates from bulk data [22] in the same tissue (Fig. 4b). We observe a similar trend across gene-uniqueness bins as was observed for the 10x datasets. Alevin demonstrates higher correlation, overall, with the bulk data, and the improvements are particularly substantial for genes that are not sequence-unique. Further, alevin is much faster and takes less memory than the other pipelines. Alevin took 17 min to process this data, which is much faster than the UMI-tools-based pipeline (∼ 3.2 h), the dropseq_utils-based pipeline (∼ 15.5 h), and even dropEst (25 min). The memory usage of alevin was 6.5 GB, which is less than half the memory usage of the closest tool (UMI-tools at 17.72 GB). The dropseq_utils-based pipeline took 25.07 GB while dropEst used 10.8 GB, which does not include the memory consumed by Cell Ranger to index the reference and align reads against it to produce the BAM file. While alevin has been primarily designed and tested with 10x data in mind, the method is generic for droplet-based tagged-end protocols, and we observe that it also seems to perform well on Drop-seq data.

## Conclusion

We present a new end-to-end pipeline for performing gene-level quantification from dscRNA-seq that is accurate, efficient, and easy to use. Our method, alevin, relies on a new formulation of the UMI resolution problem that both accounts for transcript-level constraints on how UMIs may have been generated and that allows resolving the potential origin of a UMI even when the corresponding reads map between multiple genes.

Our analyses demonstrate that, compared to Cell Ranger (and *naïve*), alevin achieves a higher accuracy, in part because of considering a substantially larger number of reads. Further, alevin is considerably faster and uses less memory than these other approaches. These speed improvements are due to a combination of the fact that alevin uses bespoke algorithms for CB and UMI edit distance computation, read mapping, and other tasks and is a unified tool for performing all of the initial processing steps, obviating the need to read and write large intermediate files on disk. These optimizations make it possible to efficiently process dscRNA-seq datasets on commodity computers reducing computational barriers to processing and re-processing of such data.

In the future, we hope to further improve the benchmarking of accuracy for single-cell quantification and barcode whitelisting approaches, as the lack of standard benchmarks makes the assessment of new methods difficult. We also hope to explore alternative cell barcode whitelisting and PUG resolution strategies—for example, adopting a generative model for PCR and sequencing error and seeking a maximum likelihood rather than maximum parsimony-based resolution of the PUGs.

Alevin is written in C++14 and is integrated into the salmon tool available at https://github.com/COMBINE-lab/salmon.

## Materials and methods

### Initial whitelisting and barcode correction

After standard quality control procedures, the first step of existing single-cell RNA-seq processing pipelines [1–3] is to extract cell barcode and UMI sequences and to add this information to the header of the sequenced read or save it in temporary files. This approach, while versatile, can create many intermediate files on disk for further processing, which can be time- and space-consuming.

Alevin begins with sample-demultiplexed FASTQ files. It quickly iterates over the file containing the barcode reads and tallies the frequency of all observed barcodes (regardless of putative errors). We denote the collection of all observed barcodes as $\mathcal {B}$. Whitelisting involves determining which of these barcodes may have derived from a valid cell. When the data has been previously processed by another pipeline, a whitelist may already be available for alevin to use. When a whitelist is not available, alevin uses a two-step procedure for calculating one. An initial draft whitelist is produced using the procedure explained below, to select CBs for initial quantification. This list is refined after per-cell-level quantification estimates are available (see “Final whitelisting (optional)” section) to produce a final whitelist.

To generate a putative whitelist, we follow the approach taken by other dscRNA-seq pipelines by analyzing the cumulative distribution of barcode frequencies and finding the knee in this curve [1, 2]. Those barcodes occurring after the knee constitute the whitelist, denoted $\mathcal {W}$. We use a Gaussian kernel to estimate the probability density function for the barcode frequency and select the local minimum corresponding to the “knee.” In the case of a user-provided whitelist, the provided $\mathcal {W}$ is used as the fixed final whitelist.

Next, we consider those barcodes in $\mathcal {E} = \mathcal {B} \setminus \mathcal {W}$ to determine, for each non-whitelisted barcode, whether (a) its corresponding reads should be assigned to some barcode in $\mathcal {W}$ or (b) this barcode represents some other type of noise or error (e.g., ambient RNA, lysed cell) and its associated reads should be discarded. The approach of alevin is to determine, for each barcode $h_{j} \in \mathcal {E}$, the set of whitelisted barcodes with which *h*_*j*_ could be associated. We call these the putative labels of *h*_*j*_—denoted as *ℓ*(*h*_*j*_). Following the criteria used by previous pipelines [1], we consider a whitelisted barcode *w*_*i*_ to be a putative label for some erroneous barcode *h*_*j*_ if *h*_*j*_ can be obtained from *w*_*i*_ by a substitution, by a single insertion (and clipping of the terminal base) or by a single deletion (and the addition of a valid nucleotide to the end of *h*_*j*_). Rather than applying traditional algorithms for computing the all-versus-all edit-distances directly, and then filtering for such occurrences, we exploit the fact that barcodes are relatively short. Therefore, we can explicitly iterate over all of the valid $w_{i} \in \mathcal {W}$ and enumerate all erroneous barcodes for which this might be a putative label. Let *Q*(*w*_*i*_,*H*) be the set of barcodes from $\mathcal {E}$ that adhere to the conditions defined above; then, for each *h*_*j*_∈*Q*(*w*_*i*_,*H*), we append *w*_*i*_ as putative label for the erroneous barcode *h*_*j*_.

Once all whitelisted barcodes have been processed, each element in $\mathcal {E}$ will have zero or more putative labels. If an erroneous barcode has more than one putative label, we prioritize substitutions over insertions and deletions. If this does not yield a single label, ties are broken randomly. If no candidate is discovered for an erroneous barcode, then this barcode is considered “noise,” and its associated reads are simply discarded. Note that, although adopted from existing methods, the alevin initial whitelisting process is designed to output a larger number of CBs.

### Mapping reads and UMI deduplication

After labeling each barcode, either as noise or as belonging to some whitelisted barcode, alevin maps the sequenced reads to the target transcriptome [10, 11]. Reads mapping to a given transcript (or multimapping to a set of transcripts) are categorized hierarchically, first based on the label of their corresponding cellular barcode, and then based on their unique molecular identifier (UMI). At this point, it is then possible to deduplicate reads based on their mapping and UMI information.

The process of read deduplication involves the identification of duplicate reads based on their UMIs and alignment positions. Most amplification occurs prior to fragmentation in library construction for 10X Chromium protocols [23]. Because of this, the alignment position of a given read is not straightforward to interpret with respect to deduplication, as the same initial unique molecule may yield reads with different alignment coordinates^2^. UMIs can also contain sequence errors. Thus, achieving the correct deduplication requires proper consideration of the available positional information and possible errors.

Our approach for handling sequencing errors and PCR errors in the UMIs is motivated by “directional” approach introduced in UMI-tools [5]. Let $\mathcal {U}_{i}$ be the set of UMIs observed for gene *i*. A specific UMI $u_{n} \in \mathcal {U}_{i}$, observed *c*_*n*_ times in gene *i*, is considered to have arisen by PCR or sequence error if there exists $u_{m} \in \mathcal {U}_{i}$ such that *d*(*u*_*n*_,*u*_*m*_)=1 and *c*_*m*_>2*c*_*n*_+1, where *d*(·,·) is the Hamming distance. Using this information, only UMIs that could not have arisen as an error under this model are retained. However, this approach may over-collapse UMIs if there exists evidence that similar UMIs (i.e., UMIs at a Hamming distance of 1 or less) may have arisen from different transcripts and, hence, distinct molecules. Moreover, this approach first discards reads that multimap to more than one read, causing it to lose a substantial amount of information before even beginning the UMI deduplication process.

As previously proposed to address the problem of cell clustering [24], an equivalence class [12, 13, 25–29] encodes some positional information, by means of encoding the set of transcripts to which a fragment is mapped. Specifically, these equivalence classes can encode constraints about which UMIs may have arisen from the same molecule and which UMIs—even if mapping to the same gene—must have derived from distinct pre-PCR molecules. This can be used to avoid over-collapsing UMI tags that are likely to result from different molecules by considering UMIs as distinct for each equivalence class. However, in its simplest form, this deduplication method is prone to reporting a considerably higher number of distinct UMIs than likely exist. This is because reads from different positions along a single transcript, and tagged with the same UMI, can give rise to different equivalence classes, so that membership in a different equivalence class is not, alone, sufficient evidence that a read must have derived from a distinct (pre-PCR) molecule. This deters us from directly using such a UMI-collapsing strategy for deriving gene-level counts, though it may be helpful for other types of analyses.

Given the shortcomings of both approaches to UMI deduplication, we propose, instead, a novel UMI resolution algorithm that takes into account transcript-level evidence when it exists, while simultaneously avoiding the problem of under-collapsing that can occur if equivalence classes are treated independently for the purposes of UMI deduplication.

### UMI resolution algorithm

A potential drawback of the gene-level deduplication is that it discards transcript-level evidence. In this case, such evidence is encoded in the equivalence classes. Thus, gene-level deduplication provides a conservative approach and assumes that it is highly unlikely for molecules that are distinct transcripts of the same gene to be tagged with a similar UMI (within an edit distance of 1 from another UMI from the same gene). However, entirely discarding transcript-level information will mask true UMI collisions to some degree, even when there is direct evidence that similar UMIs must have arisen from distinct transcripts. For example, if similar UMIs appear in transcript-disjoint equivalence classes (even if all of the transcripts labeling both classes belong to the same gene), then they *cannot* have arisen from the same pre-PCR molecule. Accounting for such cases is especially true when using an error-aware deduplication approach and as sequencing depth increases.

To perform UMI deduplication, alevin begins by constructing a *p*arsimonious *U*MI *g*raph (PUG), *G*=(*V*,*E*), for each cell, where each *v*_*i*_=(*u*,*T*_*i*_) is a tuple consisting of UMI sequence *u* and a set of transcripts $T_{i} = \{t_{i_{1}}, t_{i_{2}}, \dots, t_{i_{m}}\}$. There is a count associated with each vertex such that *c*(*v*_*i*_)=*c*_*i*_ is the number of times this UMI equivalence class pair is observed. *G* contains two types of edges: directed and bi-directed. There exists a directed edge between every pair of vertices (*v*_*i*_,*v*_*j*_) for which *c*_*i*_>2*c*_*j*_−1, |*T*_*i*_∩*T*_*j*_|>0, and *d*(umi(*v*_*i*_),umi(*v*_*j*_))=1. For every pair of vertices for which there is no directed edge, there exists a bi-directed edge if *d*(umi(*v*_*k*_),umi(*v*_*ℓ*_))≤1, and |*T*_*k*_∩*T*_*ℓ*_|>0. Once the edges of this PUG have been formed, we no longer need to consider the counts of the individual UMI equivalence class pairs.

Before proceeding further, we introduce the notion of *monochromatic arborescences* in terms of this graph *G*. We can refer to the transcript labels of each node as the potential colors of the node. Since our graph is directed, an arborescence would be a rooted tree in the graph, where each node within the arborescence has exactly one directed path reaching it from a determined root node, using edges in the arborescence. Given these definitions, a monochromatic arborescence is one where the set of colors of the nodes within the arborescence have a non-null intersection and, hence, the arborescence can be labeled using a single color. Then, for a given connected component in the graph, we can find different sets of monochromatic arborescences and, for our graph, each one represents a single pre-PCR molecule.

However, motivated by the principle of parsimony, we wish to explain the observed vertices (i.e., UMI, equivalence class pairs) via the minimum possible number of pre-PCR molecules that are consistent with the observed data. Hence, we pose this problem in the following manner. Given a graph *G*, we seek a *minimum cardinality covering by monochromatic arborescences*. In other words, we wish to cover *G* by a collection of vertex-disjoint arborescences, where each arborescence is labeled consistently by a set of transcripts, which are the pre-PCR molecule types from which its reads and UMIs are posited to have arisen. Further, we wish to cover all vertices in *G* using the minimum possible number of arborescences. Here, the graph *G* defines which UMI, read pairs can potentially be explained in terms of others (i.e., which vertices may have arisen from the same molecule by virtue of different fragmentation positions or which vertices may have given rise to other through PCR duplication with error). The decision version of this problem is NP-complete, as shown below and so, alevin employs a greedy algorithm in practice to obtain a valid, though not necessarily minimum, covering of *G*. We note that while numerous covering and packing problems related to arborescences have appeared in the literature (Bernáth and Pap [30] and references therein), to the best of our knowledge, the following problem formulation is new.

#### **Theorem 1**

Minimum cardinality covering by monochromatic arborescences is NP-complete.

#### *Proof*

Consider a reduction from dominating set. Let (*G*,*k*) be an instance of the dominating set problem where *G*=(*V*,*E*) is an undirected graph. Then, we can construct a new graph *G*^′^=(*V*,*E*^′^) such that *G*^′^ has a minimum cardinality covering by ≤*k* monochromatic arborescences if and only if *G* has a minimum dominating set of size ≤*k*. The color of an arborescence is chosen from among the intersection of the set of labels for each node it covers and, hence, is non-null. Construct *G*^′^ as follows. Convert each edge in *G* to a bi-directed edge in *G*^′^ and label each node with the union of its own label and the labels of all nodes to which it is directly connected in *G*. In other words, *T*_*i*_={*i*}∪{*j*∣{*i*,*j*}∈*E*}.

→ If *G* has a minimum dominating set of size *k*, then *G*^′^ has a minimum cardinality covering by *k* monochromatic arborescences. Every node in the original graph *G* has to be connected to at least one node in the dominating set. Due to the manner in which node labels are assigned in *G*^′^, this means that every node in *G*^′^ can be covered by an arborescence starting from a dominating set node; this arborescence is colored by the label assigned to that node. Since there are *k* nodes in the dominating set, there will be *k* monochromatic arborescences in *G*^′^, and since the *k* nodes in *G* dominate *V*, the arborescences will cover all of *V*.

← If *G*^′^ has a covering of *k* monochromatic arborescences, then *G* has a dominating set of size *k*. An arborescence is assigned a color, let us say *ℓ*_*i*_, from the intersection of the labels of the nodes it covers. Hence, the node with label *ℓ*_*i*_ in *G*^′^ has to be one of the nodes covered by this arborescence. That node connects to all the nodes in this arborescence; otherwise, they would not have shared this label. Let these nodes be selected as the dominating set of *G*. Hence, if there are *k* arborescences, there are *k* such nodes that are part of the dominating set, and because the arborescences cover all of *G*^′^, the selected nodes, likewise, dominate *G*. □

The algorithm employed by alevin works as follows. First, we note that weakly connected components of *G* can be processed independently, and so, we describe here the procedure used to resolve UMIs within a single weakly connected component—this is repeated for all such components. Let *C*=(*V*_*C*_,*E*_*C*_) denote our current component. We perform a breadth-first search starting from each vertex *v*_*i*_∈*V*_*C*_ and considering each transcript $t_{i_{j}}$ (the *j*th transcript in the equivalence class labeling vertex *v*_*i*_). We compute the size (cardinality) of the largest arborescence that can be created starting from this node and using this label to cover the visited vertices. Let $\phantom {\dot {i}\!}v_{i^{\prime }}, t_{i^{\prime }_{j^{\prime }}}$ be the vertex, transcript pair generating the largest arborescence, and let $\phantom {\dot {i}\!}a\left (v_{i^{\prime }}, t_{i^{\prime }_{j^{\prime }}}\right)$ be the corresponding arborescence. We now remove all of the vertices in $\phantom {\dot {i}\!}a\left (v_{i^{\prime }}, t_{i^{\prime }_{j^{\prime }}}\right)$, and all of their incident edges, from *C*, and we repeat the same procedure on the remaining graph. This process is iterated until all vertices of *C* have been removed. This procedure is guaranteed to select some positive order arborescence (i.e., an arborescence containing at least one node) in each iteration and hence is guaranteed to terminate after at most a linear number of iterations in the order of *C*.

After computing a covering, each arborescence is labeled with a particular transcript. However, the selected transcript may not be the unique transcript capable of producing this particular arborescence starting from the chosen root node. We can compute, for each arborescence, the *set* of possible transcript labels that could have colored it (i.e., those in the intersection of the equivalence class labels for all of the vertices in the arborescence). If the cardinality of this set is 1, then only a single transcript is capable of explaining all of the UMIs associated with this arborescence. If the cardinality of this set is > 1, then we need to determine if all transcripts capable of covering this arborescence belong to the same gene, or whether transcripts from multiple genes may, in fact, be capable of explaining the associated UMIs. In the former case, the count of pre-PCR molecules (i.e., distinct, deduplicated UMIs) associated with this uniquely selected gene is incremented by 1. In the latter case, the molecule associated with the arborescence is considered to potentially arise from any of the genes with which it could be labeled. Subsequently, an EM algorithm is used to distribute the counts between the genes. Note that other pipelines simply discard these gene-ambiguous reads and that both manners in which alevin attempts to resolve such reads (i.e., either by being selected via the parsimony condition or probabilistically allocated by the EM algorithm) are novel in the context of scRNA-seq quantification. The EM procedure we adopt to resolve ambiguous arborescences proceeds in the same manner as the EM algorithm used for transcript estimation in bulk RNA-seq data [13], with the exception that we assume the probability of generating a fragment is directly proportional to the estimated abundance, rather than the abundance divided by the effective length (i.e., we assume that, in the tagged-end protocols used, there is no length effect in the fragment generation process).

### Tier assignment

The alevin program also outputs a tier matrix, of the same dimensions as the cell gene count matrix. Within a cell, each gene is assigned one of four tiers. The first tier (assigned 0) is the set of genes that have no read evidence in this cell and are, therefore, predicted to be unexpressed (whether truly absent, or the effect of some dropout process). The rest of the tiers (1, 2, and 3) are assigned based on a graph induced by the transcript equivalence classes as follows:

1. All equivalence classes of size 1 are filtered out. The genes associated with the transcripts from these classes are assigned to tier 1.
2. For the remaining equivalence classes, of size > 1 gene, a graph *G* is constructed. The nodes in *G* are transcripts, and two nodes share an edge if their corresponding transcripts belong to a single equivalence class.
3. All the connected components in *G* are listed, and the transcript labels on the nodes mapped to their corresponding genes. If any component contains a node whose gene has previously been assigned to tier 1, that gene and all other genes in this connected component are assigned to tier 2. Hence, tier 2 contains genes whose quantification is impacted by the EM algorithm (after the UMI deduplication).
4. Genes associated with the remaining nodes in the graph are assigned to tier 3. These are genes that have no unique evidence and do not share reads (or, in fact, paths in the equivalence class graph) with another gene that has unique evidence. Hence, the EM algorithm will distribute reads between these genes in an essentially uniform manner, and their estimates are uninformative. Their abundance signifies that some genes (at least 1) in this ambiguous family are expressed, but exactly which and their distribution of abundances cannot be determined.

Alevin, optionally (using the —numCellBootstraps flag), also outputs bootstrap variance estimates for genes within each cell. These variance estimates could conceivably be used by downstream tools for dimensionality reduction, differential expression testing, or other tasks.

### Final whitelisting (optional)

Many existing tools for whitelisting CBs, such as Cell Ranger [3] and Sircel [7] perform whitelisting only once. As discussed above, both tools rely on the assumption that the number of times a CB is observed is sufficient to identify the *correct* CBs, i.e., those originating from droplets containing a cell. However, as observed by Petukhov et al. [8], there is considerable variation in sequencing depth per cell, and some droplets may contain damaged or low-quality cells. Thus, true CBs may fall below a simple knee-like threshold. Similarly, erroneous CBs may lie above the threshold. Petukhov et al. [8] proposed that instead of selecting a single threshold, one should treat whitelisting as a classification problem and segregate CBs into three regions: high quality, low quality, and uncertain/ambiguous. Here, high quality refers to the CBs which are deemed to be definitely correct, and low quality are the CBs which are deemed to most likely not arise from valid cells. A classifier can then be trained on the high- and low-quality CBs to classify the barcodes in the ambiguous region as either high or low quality. We adopt this approach in alevin, using our knee method’s cutoff to determine the ambiguous region. Specifically, we divide everything above the knee threshold into two equal regions: high-quality valid barcodes (upper-half), denoted by $\mathcal {H}$, and ambiguous barcodes (lower-half), denoted by $\mathcal {L}$. Since the initial whitelisting procedure is very liberal in selecting a threshold, most of the recoverable, low-confidence CBs tend to reside in the ambiguous region, and to learn the low-quality region, we take $n_{l} = \max (0.2 \cdot \left |\mathcal {H}\right |, 1000)$ barcodes just below the knee threshold.

In the implementation of Petukhov et al.[8], a kernel density estimation classifier was trained using features which described the number of reads per UMI, UMIs per gene, the fraction of intergenic reads, non-aligned reads, the fraction of lowly expressed genes, and the fraction of UMIs on lowly expressed genes. In addition, a maximum allowable mitochondrial read content was set for a CB to be classified as “high-quality.” Whilst these features enabled the authors to build a classifier which efficiently separated “high-quality” cells from “low-quality” cells, we believe it may be possible to improve this set of features. Specifically, most of these features would be expected to correlate with the number of reads or UMIs per CB. Thus, the classifier is biased towards attributes associated with higher read depth, when in fact one wants it to learn the feature attributes associated with high-quality cells. We therefore used a slightly different set of features, listed below, which we believe may better capture the differences between high- and low-quality cells. While these features work in general, they may not be suitable for all analyses and will have to be tweaked accordingly. We chose to use a naïve Bayes classifier to perform classification, since we observed no clear difference between multiple ML methods (not shown), and the naïve Bayes classifier yields classification probabilities which are easy to interpret. Our final set of whitelisted CBs are those classified as high confidence.

1. Fraction of reads mapped
2. Fraction of mitochondrial reads (optionally activated by —mRNA flag)
3. Fraction of rRNA reads (optionally activated by —rRNA flag)
4. Duplication rate
5. Mean gene counts post deduplication
6. The maximum correlation of gene-level quantification estimates with the high-quality CBs (optionally activated by –useCorrelation flag)

### Machine configuration and pipeline replicability

10x v2 chemistry benchmarking has been scripted using CGATCore (https://github.com/cgat-developers/cgat-core). The full pipeline and analysis are performed using Stony Brook’s seawulf cluster with 164 Intel Xeon E5–2683v3 CPUs.

For all analyses, the genome and gtf versions used for human datasets were GENCODE release 27, GRCh38.p10, and for mouse datasets were GENCODE release M16, GRCm38.p5. All transcriptome files were generated using these with “rsem-prepare-reference.”

*Cell Ranger (v2.2.0):* The following additional flags were used, as recommended by the Cell Ranger guidelines: —nosecondary —expect-cells NumCells, where NumCells is 10,000 for PBMC 8k and Neurons 9k, 5,000 for PBMC 4k, and 2,000 for Neurons 2k and Neurons 900.

*Alevin (v0.13.0):* Run with default parameters with the Chromium protocol and —keepDuplicates flags and the -lISR to specify strandedness. The mRNA and rRNA lists were obtained from the relevant annotation files and passed as input. Experiments on v1 chemistry can be run using the same flags but with the —gemcode protocol flag. Alevin also supports 10x v3 chemistry via the command-line flag —chromiumV3.

*STAR (v2.6.0a):* The following flag was used, as recommended by the guidelines of UMI-tools: —outFilterMultimapNmax 1

*featureCounts (v1.6.3):* This was run to obtain an output BAM file and with stranded input (-s 1).

*UMI-tools (v0.5.4):* The extract command was used to get the CBs/UMIs, when provided with an external CB whitelist and attach it to the corresponding reads. The following flags were used in the count command to obtain the per-cell gene count matrix: —gene-tag=XT —wide-format-cell-counts

*DropEst (v0.8.5)*: This was run with the default parameters on the 10x BAM files, and the predicted cell counts from Cell Ranger were used as input.

*Dropseq utils (v2.0.0):* All the commands were run as recommended by the authors in the tool’s manual.

The bulk datasets were quantified using Bowtie2 and RSEM, run as follows:

*Bowtie2 (v2.3.4.3):* The following flags were used, as recommended in the guidelines of RSEM: —sensitive —dpad 0 —gbar 99999999 —mp 1,1 —np 1 —score-min L,0,-0.1 —no-mixed —no-discordant

*RSEM (v1.3.1):* Run with default parameters.

## Additional file

Additional file 1 Supplementary material for alevin efficiently estimates accurate gene abundances from dscRNA-seq data. Includes supplementary figures. (PDF 669 kb)

## Footnotes

1 Note that we were not able to run the dropEstr Bayesian correction method and the results presented are after running just the dropEst pipeline [31].

2 We note that whether the majority of amplification occurs pre- or post-fragmentation can be protocol specific and can suggest different strategies for UMI deduplication. Here, we are primarily concerned with the 10X Chromium protocols, dominated by pre-fragmentation amplification. However, the method we propose for UMI deduplication can be applied to other protocols as well.

## References

[1] Macosko EZ, Basu A, Satija R, Nemesh J, Shekhar K, Goldman M (2015). Highly parallel genome-wide expression profiling of individual cells using nanoliter droplets. Cell.

[2] Klein AM, Mazutis L, Akartuna I, Tallapragada N, Veres A, Li V (2015). Droplet barcoding for single-cell transcriptomics applied to embryonic stem cells. Cell.

[3] Zheng GX, Terry JM, Belgrader P, Ryvkin P, Bent ZW, Wilson R (2017). Massively parallel digital transcriptional profiling of single cells. Nat Commun.

[4] Svensson V, Natarajan KN, Ly LH, Miragaia RJ, Labalette C, Macaulay IC (2017). Power analysis of single-cell RNA-sequencing experiments. Nat Methods.

[5] Smith T, Heger A, Sudbery I (2017). UMI-tools: modeling sequencing errors in Unique Molecular Identifiers to improve quantification accuracy. Genome Res.

[6] Zhao L, Liu Z, Levy SF, Wu S (2017). Bartender: a fast and accurate clustering algorithm to count barcode reads. Bioinformatics.

[7] Tambe A, Pachter L (2019). Barcode identification for single cell genomics. BMC Bioinformatics.

[8] Petukhov V, Guo J, Baryawno N, Severe N, Scadden DT, Samsonova MG, Kharchenko PV (2018). dropEst: pipeline for accurate estimation of molecular counts in droplet-based single-cell RNA-seq experiments. Genome Biol.

[9] Tian L, Su S, Dong X, Amann-Zalcenstein D, Biben C, Seidi A (2018). scPipe: a flexible R/Bioconductor preprocessing pipeline for single-cell RNA-sequencing data. PLoS Comput Biol.

[10] Srivastava A, Sarkar H, Gupta N, Patro R (2016). RapMap: a rapid, sensitive and accurate tool for mapping RNA-seq reads to transcriptomes. Bioinformatics.

[11] Sarkar H, Zakeri M, Malik L, Patro R (2018). Towards selective-alignment: bridging the accuracy gap between alignment-based and alignment-free transcript quantification. Proceedings of the 2018 ACM International Conference on Bioinformatics, Computational Biology, and Health Informatics. BCB ’18.

[12] Turro E, Su SY, Gonçalves Â, Coin LJ, Richardson S, Lewin A (2011). Haplotype and isoform specific expression estimation using multi-mapping RNA-seq reads. Genome Biol.

[13] Patro R, Duggal G, Kingsford C (2017). Salmon provides fast and bias-aware quantification of transcript expression. Nat Methods.

[14] Dobin A, Davis CA, Schlesinger F, Drenkow J, Zaleski C, Jha S (2013). STAR: ultrafast universal RNA-seq aligner. Bioinformatics.

[15] Liao Y, Smyth GK, Shi W (2013). featureCounts: an efficient general purpose program for assigning sequence reads to genomic features. Bioinformatics.

[16] Li B, Dewey CN (2011). RSEM: accurate transcript quantification from RNA-Seq data with or without a reference genome. BMC Bioinformatics.

[17] Langmead B, Salzberg SL (2012). Fast gapped-read alignment with Bowtie 2. Nat Methods.

[18] Robert C, Watson M (2015). Errors in RNA-Seq quantification affect genes of relevance to human disease. Genome Biol.

[19] Han X, Wang R, Zhou Y, Fei L, Sun H, Lai S (2018). Mapping the mouse cell atlas by Microwell-seq. Cell.

[20] Richter F, Meurers BH, Zhu C, Medvedeva VP, Chesselet MF (2009). Neurons express hemoglobin *α*-and *β*-chains in rat and human brains. J Comp Neurol.

[21] Nakaya HI, Wrammert J, Lee EK, Racioppi L, Marie-Kunze S, Haining WN (2011). Systems biology of vacination for seasonal influenza in humans. Nat Immunol.

[22] Grant GR, Farkas MH, Pizarro AD, Lahens NF, Schug J, Brunk BP (2011). Comparative analysis of RNA-Seq alignment algorithms and the RNA-Seq unified mapper (RUM). Bioinformatics.

[23] 10x-Genomics Single-Cell 3’-V2 Kit. 2018. https://teichlab.github.io/scg_lib_structs/data/CG000108_AssayConfiguration_SC3v2.pdf.

[24] Ntranos V, Kamath GM, Zhang JM, Pachter L, David NT (2016). Fast and accurate single-cell RNA-seq analysis by clustering of transcript-compatibility counts. Genome Biol.

[25] Mezlini AM, Smith EJ, Fiume M, Buske O, Savich GL, Shah S (2013). iReckon: simultaneous isoform discovery and abundance estimation from RNA-seq data. Genome Res.

[26] Patro R, Mount SM, Kingsford C (2014). Sailfish enables alignment-free isoform quantification from RNA-seq reads using lightweight algorithms. Nat Biotechno.

[27] Bray NL, Pimentel H, Melsted P, Pachter L (2016). Near-optimal probabilistic RNA-seq quantification. Nat Biotechnol.

[28] Zhang Z, Wang W (2014). RNA-Skim: a rapid method for RNA-Seq quantification at transcript level. Bioinformatics.

[29] Ju CJT, Li R, Wu Z, Jiang JY, Yang Z, Wang W (2017). Fleximer: accurate quantification of, RNA-Seq via variable-length k-mers. Proceedings of the 8th ACM International Conference on Bioinformatics, Computational Biology, and Health Informatics. ACM-BCB ’17.

[30] Bernáth A, Pap G (2011). Covering minimum cost arborescences.

[31] Pipeline for initial analysis of droplet-based single-cell RNA-seq data. 2018. https://github.com/hms-dbmi/dropEst. Accessed: 19 Oct 2018.

[32] Poldrack RA, Laumann TO, Koyejo O, Gregory B, Hover A, Chen MY (2015). Long-term neural and physiological phenotyping of a single human. Nat Commun.

[33] Dvinge H, Ries RE, Ilagan JO, Stirewalt DL, Meshinchi S, Bradley RK (2014). Sample processing obscures cancer-specific alterations in leukemic transcriptomes. Proc Natl Acad Sci.

[34] Bouquet J, Soloski MJ, Swei A, Cheadle C, Federman S, Billaud JN (2016). Longitudinal transcriptome analysis reveals a sustained differential gene expression signature in patients treated for acute Lyme disease. MBio.

[35] Shen Y, Lu Bu RL, Tian F, Lu N, Ge Q (2017). Screening effective differential expression genes for hepatic carcinoma with metastasis in the peripheral blood mononuclear cells by RNA-seq. Oncotarget.

[36] Schmitt BM, Rudolph KL, Karagianni P, Fonseca NA, White RJ, Talianidis I, et al.High-resolution mapping of transcriptional dynamics across tissue development reveals a stable mRNA–tRNA interface. Genome Res. 2014:gr–176784. 10.1101/gr.176784.114 PMC4216921 25122613

[37] Saito Y, Miranda-Rottmann S, Ruggiu M, Park CY, Fak JJ, Zhong R (2016). NOVA2-mediated RNA regulation is required for axonal pathfinding during development. Elife.

[38] Fratta P, Sivakumar P, Humphrey J, Lo K, Ricketts T, Oliveira H (2018). Mice with endogenous TDP-43 mutations exhibit gain of splicing function and characteristics of amyotrophic lateral sclerosis. EMBO J.

[39] Srivastava A, Malik L, Smith T, Sudbery I, Patro R. Alevin efficiently estimates accurate gene abundances from dscRNA-seq data: source Code: Zenodo; 2019. Available from: https://zenodo.org/record/2583275. Accessed 4 Mar 2019. 10.1186/s13059-019-1670-y PMC6437997 30917859

[40] Srivastava A, Malik L, Smith T, Sudbery I, Patro R. Alevin efficiently estimates accurate gene abundances from dscRNA-seq data: github; 2019. Available from: https://github.com/COMBINE-lab/salmon. Accessed 4 Mar 2019. 10.1186/s13059-019-1670-y PMC6437997 30917859

[41] Srivastava A, Malik L, Smith T, Sudbery I, Patro R. Alevin efficiently estimates accurate gene abundances from dscRNA-seq data: data: Zenodo; 2019. Available from: https://zenodo.org/record/2583228. Accessed 4 Mar 2019. 10.1186/s13059-019-1670-y PMC6437997 30917859

[42] 10x-Genomics v2 Chemistry Data. 2018. https://support.10xgenomics.com/single-cell-gene-expression/datasets.

