# Supplementary material for: Alevin efficiently estimates accurate gene abundances from dscRNA-seq data
Source: Genome Biol. 2019 Mar 27;20:65. doi: 10.1186/s13059-019-1670-y (PMC6437997; doi:10.1186/s13059-019-1670-y)
Supplement: Supplementary file 1 — Supplementary material for alevin efficiently estimates accurate gene abundances from dscRNA-seq data. Includes supplementary figures. (PDF 669 kb) [file 13059_2019_1670_MOESM1_ESM.pdf]

Supplementary Material for Alevin efficiently estimates accurate gene abundances from dscRNA-seq data

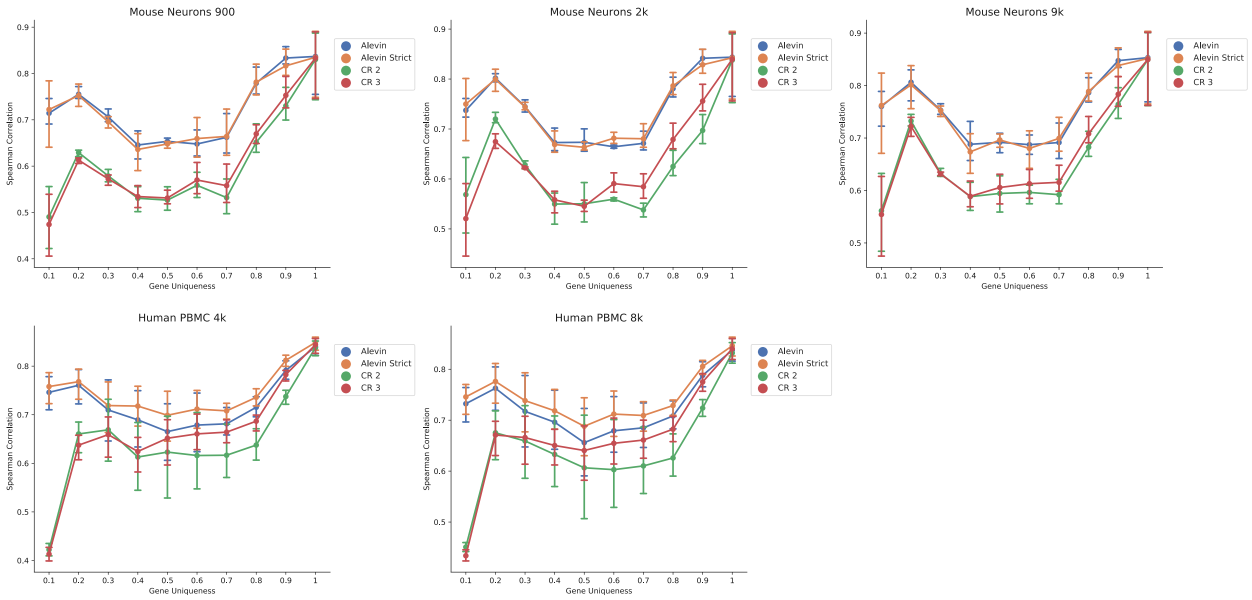

Figure S1: The Spearman correlation between quantification estimates from different runs of alevin and Cell-Ranger. Note that two different versions Cell-Ranger were run with the default parameters and alevin strict refers to the same version of alevin run with `--minScoreFraction` set to 0.95 and `--consensusSlack` set to 0.99. These parameters in alevin make the mapping filter strict and allows fewer spurious mappings.

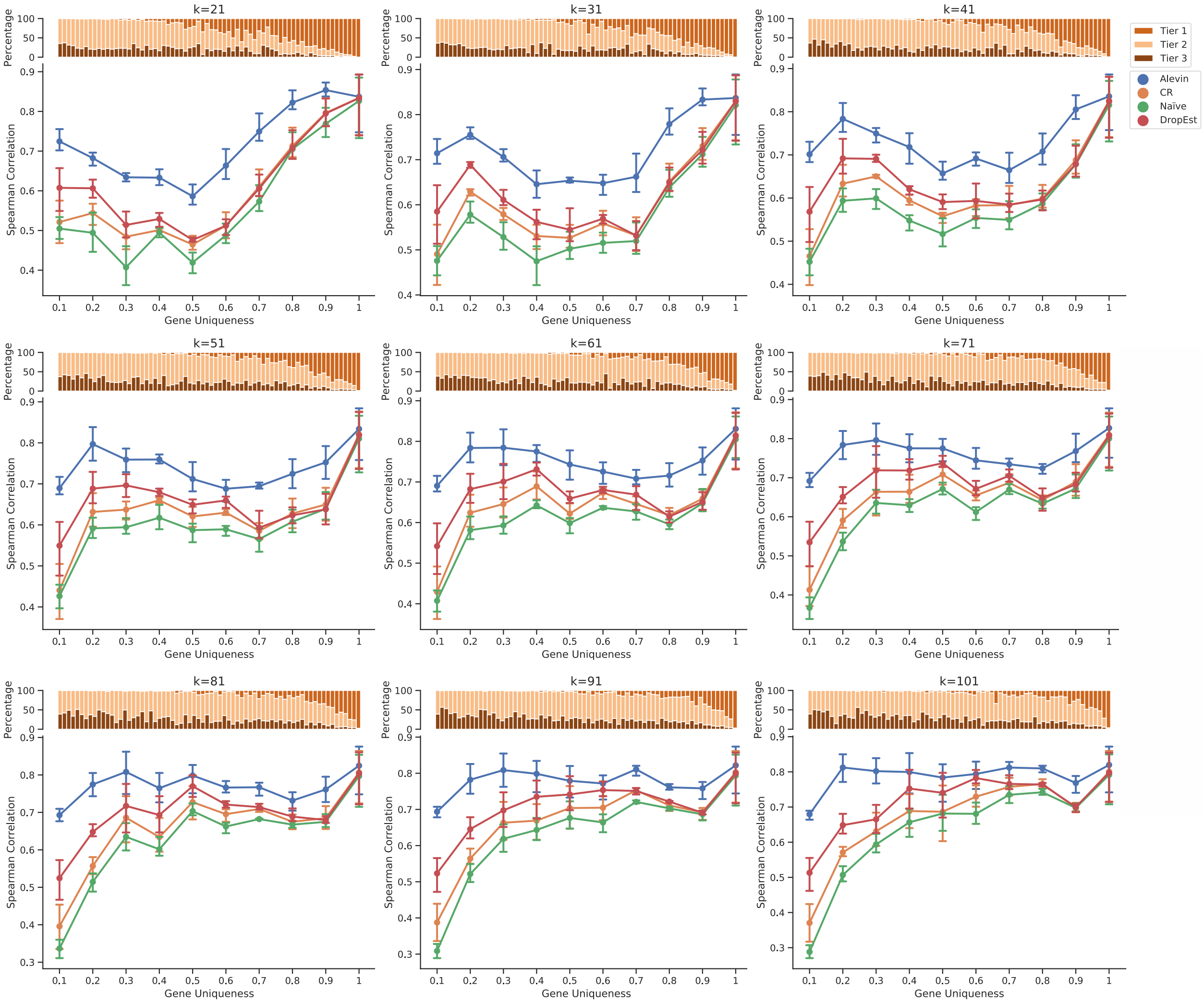

Figure S2: Correlation plots for the mouse neuronal 900 dataset using different values of the k-mer size (k) to calculate gene uniqueness.

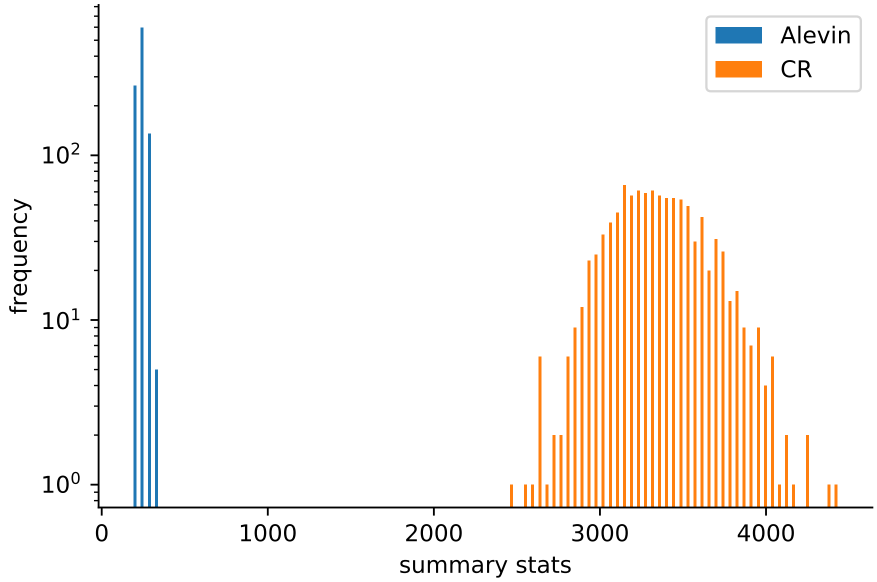

Figure S3: The histogram is the result of taking 1000 samples of 100 cells each from the mouse neuronal 900 dataset, and looking at the sum of absolute differences when quantifying the data under the varying reference genomes (mouse vs. mouse and human combined).
